# Active site competition is the mechanism for the inhibition of lipoprotein-associated phospholipase A_2_ by detergent micelles or lipoproteins and for the efficacy reduction of darapladib

**DOI:** 10.1038/s41598-020-74236-0

**Published:** 2020-10-14

**Authors:** Shaoqiu Zhuo, Chong Yuan

**Affiliations:** 1 Diazyme Laboratories, Inc, 12889 Gregg Ct., Poway, CA 92064 USA; 2 Present Address: Bayer HealthCare, 800 Dwight Way, Berkeley, CA 94710 USA

**Keywords:** Biochemistry, Drug discovery, Medical research

## Abstract

Lipoprotein associated phospholipase A_2_ (Lp-PLA_2_) has been characterized for its interfacial activation as well as inhibition by detergent micelles and lipoprotein particles. The enzyme has been shown to bind on the surfaces of hydrophobic aggregates, such as detergent micelles, lipoprotein particles and even polystyrene latex nanobeads. Binding to hydrophobic aggregates stimulates the activity of Lp-PLA_2_ but may not be the necessary step for catalysis. However, at higher concentrations, detergent micelles, latex nanobeads or lipoprotein particles inhibit Lp-PLA_2_ possibly by blocking the access of substrates to the active site. The competition mechanism also blocks inhibitors such as darapladib binding to Lp-PLA_2_ and reduces the efficacy of the drug. Darapladib has very low solubility and mainly exists in solutions as complexes with detergents or lipoprotein particles. The inhibition of Lp-PLA_2_ by darapladib is dependent on many factors such as concentrations of detergents or lipoproteins, incubation time, as well as the order of mixing reaction components. The in vitro Lp-PLA_2_ activity assays used in clinical studies may not accurately reflect the residual Lp-PLA_2_ activity in vivo. Darapladib has been found mainly bound on HDL and albumin when it is incubated with human serum. However, Lp-PLA_2_ is more sensitive to darapladib when bound on LDL and relatively resistant to darapladib when bound on HDL. Therefore, high cholesterol levels may decrease the efficacy of darapladip and cause the drug to be less effective in high risk patients. Our study will help to design better inhibitors for Lp-PLA_2_. The discoveries also contribute to understanding the mechanism of interfacial activation and inhibition for Lp-PLA_2_ and provide a new concept for researchers in building better kinetic model for interfacial enzymes.

## Introduction

Cholesterol and triglycerides are hydrophobic nutrition and cell structural compounds which are necessary for the function of animal cells and need to be transported between different cell types and tissues through the aqueous circulation system. Lipoproteins are complex particles which facilitate the transportation of hydrophobic compounds in the aqueous milieu. Lipoprotein particles have a central core containing cholesterol esters and triglycerides surrounded by a spherical monolayer of phospholipids incorporated with free cholesterol and apolipoproteins^1^. Functions of apolipoproteins include serving a structural role and guiding the formation of lipoproteins, acting as ligands for lipoprotein receptors and serving as activators or inhibitors of enzymes involved in the metabolism of lipoproteins^2^. Different types of lipoprotein particles carry different apolipoproteins and have different size and density^1^. Low density lipoprotein (LDL) and high density lipoprotein (HDL) are two of the major lipoproteins in the human circulatory system^2^. LDL delivers triglyceride and cholesterol to peripheral tissues and HDL transports triglyceride and cholesterol back to liver (reverse cholesterol efflux)^2^. Higher levels of LDL cholesterol (“bad cholesterol”) correlate with atherosclerosis and higher risk of developing coronary artery diseases (CAD) whereas higher levels of HDL cholesterol (“good cholesterol”) show an inverse relationship with CAD risk^2^. Atherosclerosis involves LDL and leukocyte infiltration into the intima (inner layer endothelial lining) of artery and formation of plaques, resulting in the hardening of arteries and thrombosis^3,4^.

Apart from transporting lipids, lipoproteins are also thought to serve as a modality for intra-organismal protein transfer, shipping proteins with important roles in inflammation and thrombosis from the sites of synthesis to effector locations^5^. Lipoprotein associated phospholipase A_2_ (Lp-PLA_2_), also known as plasma platelet-activating factor acetylhydrolase (PAF-AH), is one such enzyme, which has been linked to vascular inflammation and atherosclerosis^6–9^. Lp-PLA_2_ is synthesized and secreted by macrophages, T-lymphocytes and other inflammatory cells but it mainly associates with LDL and HDL in an average ratio about 3:1 respectively^10–13^. Lp-PLA_2_ has been shown to be present on rapture-prone plaques but not on stable plaques and, therefore, suspected to have a role in thrombosis^13,14^. However, a potent inhibitor of Lp-PLA_2_, darapladib, failed to show effectiveness in two large pivotal clinical trials^15,16^. In patients with stable coronary heart disease, darapladib did not significantly reduce the risk of the primary composite end point of cardiovascular death, myocardial infarction, or stroke although it did appear to arrest the growth of the necrotic core of atherosclerotic arteries, the area prone to rupture, in a phase 2 clinical trial^17,18^. The variability of plaque penetration by the drug was suggested by the clinical team as one of the possible causes responsible for the absence of efficacy of darapladib^15^. Two Lp-PLA_2_ activity assays, one colorimetric and another radiometric, were used during the clinical studies to assess the residual enzyme activity and it was claimed that there were approximately 66% reduction of Lp-PLA_2_ activity when darapladib was dosed at 160 mg daily for 12 weeks^15,16^. The radiometric assay has been used in initial clinical studies, whereas the more practical colorimetric assay was introduced in larger clinical trials^19^. Although two assays are correlated well, these assays report numerically different inhibition of Lp-PLA_2_ activity with darapladib administration^19^. The main difference is that the radiometric assay contains no detergents and the colorimetric assay contains 5–10 mM CHAPS. Characterization of the activation and inhibition of recombinant Lp-PLA_2_ in detergent micelles and studying the inhibition of LDL and HDL associated Lp-PLA_2_ indicate that in vivo inhibition of Lp-PLA_2_ may be more complicated than initially believed. The colorimetric activity assays used in the clinical studies may not reflect the real residual activity of Lp-PLA_2_ in human plasma. There is a possibility that the lack of clinical benefit for darapladib may also be due to its low efficacy in the inhibition of Lp-PLA_2_. Thus, the question is whether the clinical studies definitively inform upon the role that Lp-PLA_2_ may play in atherogenesis. Understanding the mechanism of Lp-PLA_2_ distribution, activation and inhibition in LDL and HDL lipoproteins may provide insights for their roles in CAD either as pathological causes or diagnostic markers.

## Results

### Effects of detergent micelles, latex beads and lipoproteins on the activation and inhibition of recombinant Lp-PLA_2_

**Figure 1:**
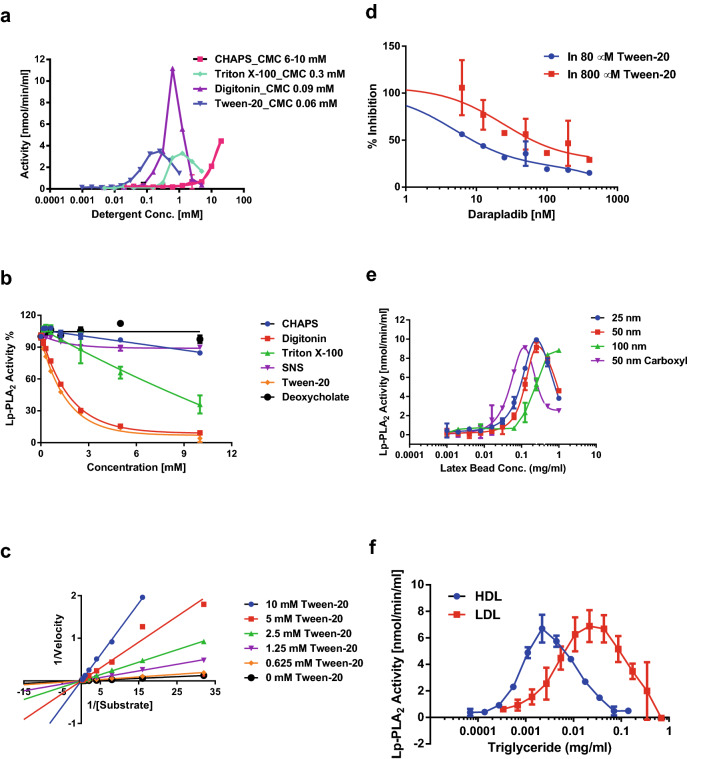
Effects of detergents, latex nanobeads and lipoprotein particles on the activation and inhibition of Lp-PLA_2_. (**a**) Lp-PLA_2_ activity in TBS, pH 7.4, containing 10 µM 14:0 NPS-PC were followed with the titration of CHAPS, Triton X-100, digitonin and Tween-20 respectively as described in Experimental Procedures. (**b**) Lp-PLA_2_ activity in 540 µM 14:0 NPS-PC were followed with the titration of CHAPS, digitonin, Triton X-100 and SNS respectively as described in Experimental Procedures. (**c**) Lineweaver–Burk plots for competitive inhibition of Lp-PLA_2_ by different concentrations of Tween-20 (TW20) as indicated. (**d**) Inhibition of Lp-PLA_2_ by darapladib in presence of 80 µM and 800 µM of Tween-20. Assay was carried out in 100 mM HEPES buffer, pH 7.5, containing 0.54 mM 14:0 NPS-PC, 4 mM EDTA and 10 mM SNS. (**e**) Lp-PLA_2_ activity in TBS, pH 7.4, containing 10 µM 14:0 NPS-PC were followed with the titration of four different polystyrene nanobeads. (**f**) Lp-PLA_2_ activity in TBS, pH 7.4, containing 10 µM 14:0 NPS-PC were followed with the titration of Lp-PLA_2_-depleted HDL and LDL as indicated.

To investigate the effects of detergents on the activity of Lp-PLA_2_, recombinant Lp-PLA_2_ was incubated with low concentrations (10 µM or lower) of artificial substrate, 1-myristoyl-2-(4-nitrophenylsuccinyl)-sn-glycero-3-phosphocholine (14:0 NPS-PC), at pH 7.4. Figure 1a show the activation and inhibition of Lp-PLA_2_ by four detergents with different critical micelle concentration (CMC). Like other lipases, Lp-PLA_2_ has very low activity towards water soluble substrates in the absence of a detergent. When titrated with a detergent, starting around the CMC of each detergent, the activity of the recombinant Lp-PLA_2_ was suddenly enhanced and then gradually decreased back to the baseline with increasing concentration of the detergent. The activation and subsequent inhibition of recombinant Lp-PLA_2_ activity resulted in the formation of a peak in the titration curves of each detergent. The formation of the peak correlated to the CMC of each detergent. Inhibition of Lp-PLA_2_ activity was also observed in reactions with higher concentrations (0.54 mM) of the substrate, 14:0 NPS-PC, for detergents with lower CMC such as Tween-20, digitonin and Triton X-100 but not for detergents with higher CMC such as CHAPS, deoxycholate and SNS (Fig. 1b). Studying the inhibition of Lp-PLA_2_ by Tween-20 revealed that it was competitive between the substrate and detergent (Fig. 1c). The Michaelis–Menten kinetic parameters of the hydrolysis reaction of 14:0 NPS-PC by Lp-PLA_2_ were also compared between the presence and absence of 10 mM CHAPS (Table 1). The difference between two V_max_ values was within the experimental error, but the difference between two K_m_ values was about three times of the experimental error. Therefore, K_m_ was affected in the presence of 10 mM CHAPS whilst the V_max_ was constant. Detergents were also found to affect the efficacy of darapladib on the inhibition of Lp-PLA_2_. The inhibitory effects of darapladib were significantly reduced when the concentration of Tween-20 was increased tenfold (Fig. 1d). The IC_50_ of darapladib was about 10 nM in 80 µM Tween-20 and 80 nM in 800 µM Tween-20.

**Table 1:** Michaelis–Menten kinetic parameters of hydrolysis reaction of 14:0 NPS-PC by Lp-PLA_2_.

	With 10 mM CHAPS	Without CHAPS
V_max_ [nmol/min/ml]	187 ± 2	191 ± 5
K_m_ [µM]	238.4 ± 11.7	187.1 ± 18.5

To further investigate the nature of the activation and inhibition of Lp-PLA_2_ under the condition of low substrate concentration, polystyrene latex nanobeads were used to mimic the roles of detergent micelles. Figure 1e shows that latex nanobeads can also activate and inhibit Lp-PLA_2_ activity under low 14:0 NPS-PC concentration in the same way as detergent micelles. Latex nanobeads with diameter 25–50 nm had the maximum activation of Lp-PLA_2_ at about 0.25 mg/ml (0.025%) concentration and became inhibitory at higher concentration. Activation of Lp-PLA_2_ by 100 nm latex nanobeads was less potent and no inhibition was observed at up to 1 mg/ml (0.1%) of latex nanobeads. Activation and inhibition of Lp-PLA_2_ by 50 nm latex nanobeads were about twice potent if the beads carrying negative charges. Latex nanobeads with diameter 25 nm have the similar sizes as LDL particles except latex nanobeads are spherical and LDL may be discoidal^20^. To test if binding to HDL and LDL can activate Lp-PLA_2_, human HDL and LDL devoid of Lp-PLA_2_ were incubated with 0.2 µg/ml recombinant Lp-PLA_2_ and 10 µM 14:0 NPS-PC in PBS, pH 7.4, as indicated in Fig. 1f. HDL and LDL were both found to activate at lower concentration and inhibit at higher concentration for recombinant Lp-PLA_2_ (Fig. 1f).

### Effects of detergent micelles on the activation and inhibition of lipoprotein-bound Lp-PLA_2_

**Figure 2:**
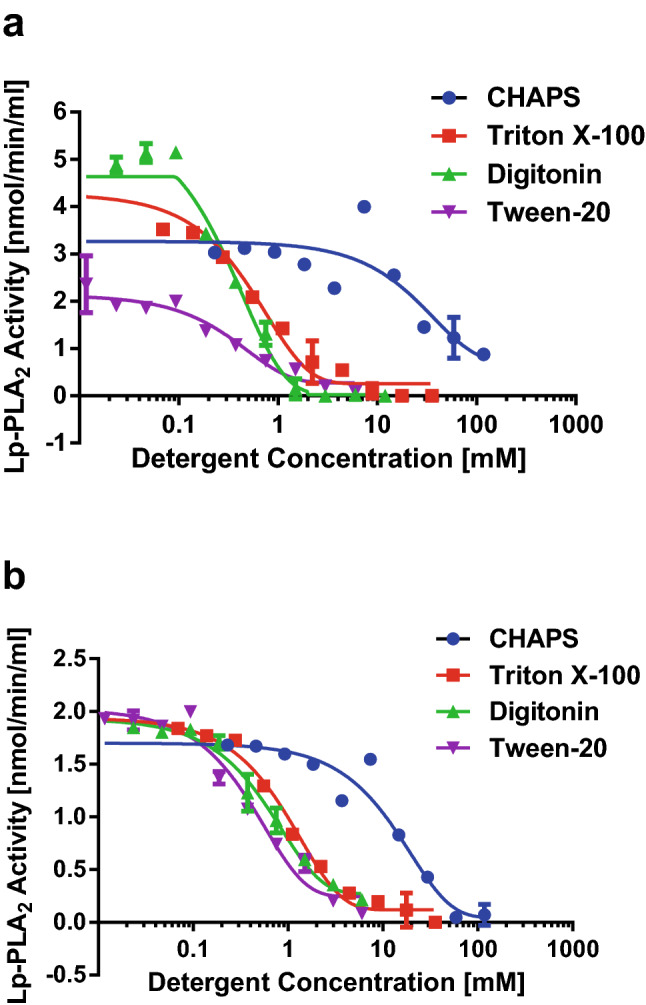
Effects of detergents on the activity of Lp-PLA_2_ associated with LDL and HDL. (**a**) Changes of LDL Lp-PLA_2_ activity with titration of detergents. (**b**) Changes of HDL Lp-PLA_2_ activity with titration of detergents. Lp-PLA_2_ activity of 20 µl LDL at 1 mg/ml or HDL at 0.15 mg/ml cholesterol were determined in final volume of 130 µl containing 10 µM of 14:0 NPS-PC and detergents as indicated.

The effects of detergents on the activity of Lp-PLA_2_ bound on HDL and LDL were also investigated. Under similar conditions as above, titration of detergents did not show clear activation peak for Lp-PLA_2_ bound on LDL (Fig. 2a) and HDL (Fig. 2b) because the lipoprotein bound enzyme is already in activated state. There were small Lp-PLA_2_ activity spikes observed for CHAPS in both HDL and LDL and for digitonin in LDL. However, increase of detergent concentration did inhibit the enzyme with the IC_50_ proportional roughly to the CMC of each detergent.

### Kinetic difference for the hydrolysis of 14:0 NPS-PC by Lp-PLA_2_ in the presence and absence of detergents

**Figure 3:**
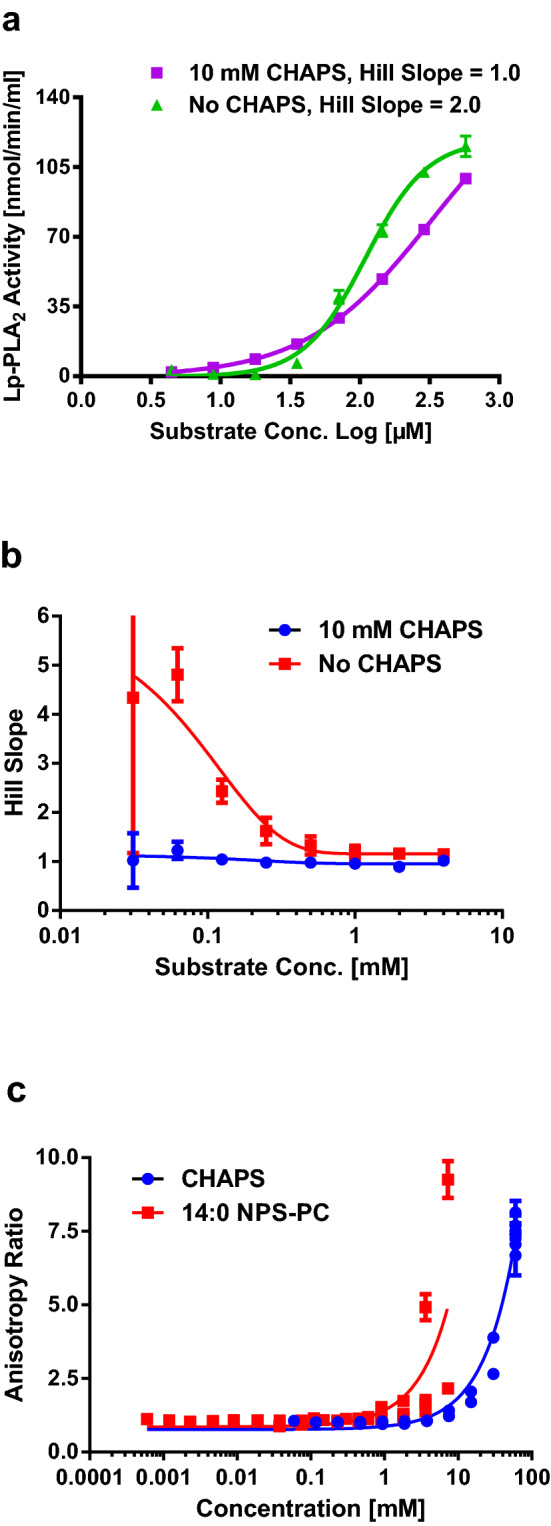
Kinetic model fitting and Hill slope of recombinant Lp-PLA_2_ in the hydrolysis of 14:0 NPS-PC. (**a**) Fitting of kinetic model for hydrolysis of 14:0 NPS-PC in the absence and presence of 10 mM CHAPS. (**b**) Change of Hill slope in kinetic fitting with concentrations of substrate in the absence and presence of 10 mM CHAPS for monomeric recombinant Lp-PLA_2_. Details are in Experiment Procedures. (**c**) Determination of CMC for 14:0 NPS-PC and CHAPS by anisotropy of polarized fluorescence using fluorescein as probe. Details are in Experiment Procedures.

To further explore the kinetic difference of Lp-PLA_2_ in the presence and absence of detergents, the hydrolysis reaction was carried out with and without 10 mM CHAPS (Fig. 3a). In the presence of 10 mM CHAPS, the reaction obeyed Michaelis–Menten kinetics but it did not do so in the absence of 10 mM CHAPS. Under these conditions, the Hill slope of the substrate titration curve was approximately 1.0 in the presence of 10 mM CHAPS and approximately 2.0 in the absence of 10 mM CHAPS. The Hill slope values were dependent on the concentration range of the substrate in the reaction. The lower of the concentration of substrate, the higher was the Hill slope values (Fig. 3b). The Hill slope decreased with the increase of substrate concentration and approached 1.0 at the substrate concentration approximately 0.5–1 mM. However, in the presence of 10 mM CHAPS, the Hill slope of substrate titration was kept at 1.0 and independent of the substrate concentration (Fig. 3b). The CMC of 14:0 NPS-PC was determined by the method of polarized fluorescence^21^ using fluorescein as the probe in TBS, pH 7.4. When the concentration of lipid or detergent approaches its CMC, the fluorescent anisotropy ratio will increase due to the formation of micelles carrying fluorescein. Figure 3c shows that the CMC of 14:0 NPS-PC is about 0.5 – 1 mM and the obtained CMC of CHAPS (control) by the same method is about 5–10 mM, which is in agreement with the literature. Interestingly, the determined CMC value of 14:0 NPS-PC is close to the substrate concentration (0.5–1 mM, Fig. 1b) when the Hill Slope approaches 1.0 and much higher than the substrate concentration when the reaction rate starts to increase along the sigmoidal curve (Fig. 3a), which is around 20–30 µM (log1.2–log1.5).

### Inhibition of lipoprotein-bound Lp-PLA_2_ by lipoprotein particles

**Figure 4:**
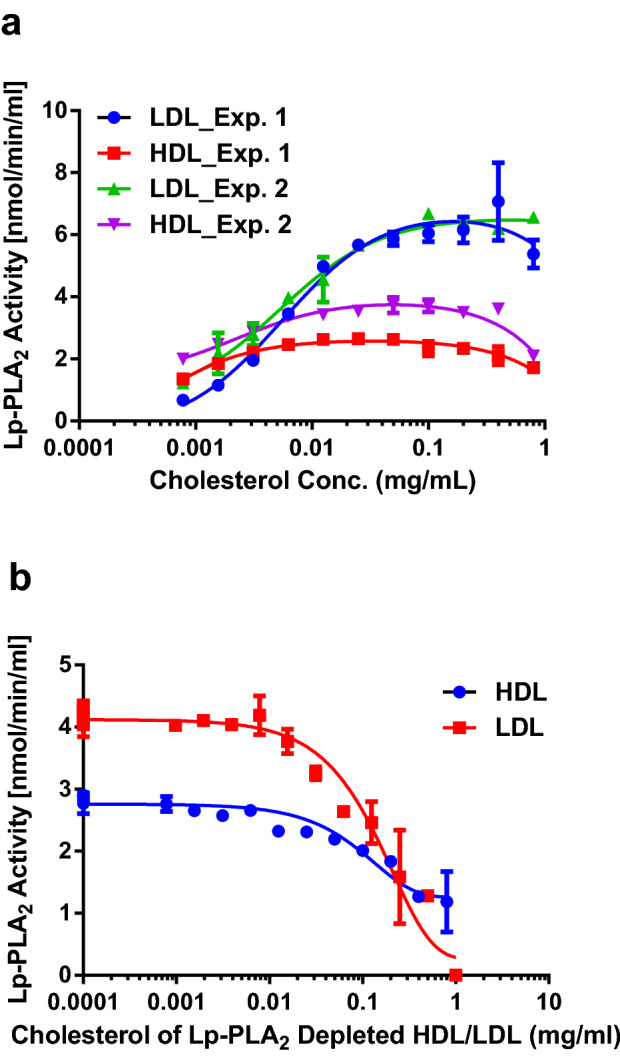
The relationship between Lp-PLA_2_ activity and lipoprotein concentration. (**a**) Lp-PLA_2_ activity by incrementation of lipoprotein. Lp-PLA_2_ activity of 20 µl lipoproteins (LDL and HDL) were determined in 10 µM of 14:0 NPS-PC with increasing concentration of lipoproteins as indicated. Lipoprotein concentration is expressed as cholesterol content. (**b**) Inhibition of Lp-PLA_2_ activity by Lp-PLA_2_-depleted lipoproteins. Lp-PLA_2_ activity of 20 µl LDL at 0.4 mg/ml or HDL at 0.04 mg/ml were determined in 10 µM of 14:0 NPS-PC with increasing concentrations of Lp-PLA_2_-depleted lipoproteins as indicated. Lipoprotein concentration is expressed as cholesterol content. Lp-PLA_2_ in lipoproteins was depleted by incubation with Pefabloc SC as descripted in Experimental Procedure.

The relationship between substrate and lipoprotein-bound Lp-PLA_2_ was investigated by titration of lipoprotein under limiting substrate concentration (10 µM 14:0 NPS-PC). Lp-PLA_2_ activity increased with lipoprotein concentration at the initial phase due to the increase of Lp-PLA_2_ concentration and achieved a plateau value of at 0.008 mg/ml for HDL and 0.06 mg/ml for LDL respectively (Fig. 4a). The activity of Lp-PLA_2_ remained essentially constant as each lipoprotein’s concentration increased up to 1 mg/ml indicating that the concentration of 14:0 NPS-PC was in limiting for the reaction rate. To further explore the effects of lipoproteins on the hydrolysis reaction of Lp-PLA_2_, HDL at 0.04 mg/ml and LDL at 0.4 mg/ml were assayed in TBS, pH 7.4, containing 10 µM of 14:0 NPS-PC and different concentrations of Lp-PLA_2_-depleted HDL and LDL. In contrast to adding Lp-PLA_2_-carrying lipoproteins, the addition of Lp-PLA_2_-depleted lipoprotein particles quickly suppressed the hydrolysis of 14:0 NPS-PC (Fig. 4b).

### Inhibition of Lp-PLA_2_ bound on lipoprotein particles by darapladib

**Figure 5:**
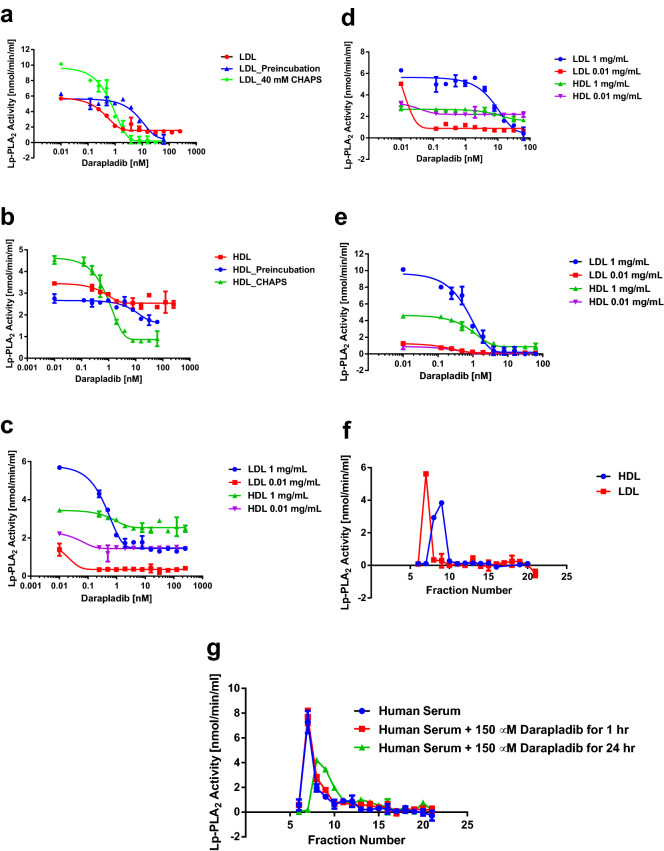
Inhibition of Lp-PLA_2_ activity associated with LDL and HDL by darapladib. (**a**) Inhibition of LDL Lp-PLA_2_ activity under three different conditions: (1) no preincubation with darapladib, (2) preincubated with darapladib for 30 min at ambient temperature and (3) preincubated with darapladib for 30 min at ambient temperature in the presence of 40 mM CHAPS. (**b**) Inhibition of HDL Lp-PLA_2_ activity under the same conditions as in (a). (**c**) Comparing the inhibition of Lp-PLA_2_ activity associated with LDL and HDL by darapladib under different concentration of lipoproteins as indicated without preincubation. (**d**) Comparing the inhibition of Lp-PLA_2_ activity associated with LDL and HDL by darapladib under different concentration of lipoproteins as indicated with preincubation at ambient temperature for 30 min before adding 14:0 NPS-PC. (**e**) Comparing the inhibition of Lp-PLA_2_ activity associated with LDL and HDL by darapladib under different concentration of lipoproteins as indicated with preincubation in the presence of 40 mM CHAPS. (**f**) Fractionation of purified HDL and LDL by TSKgel G3000SW_XL_ column in TBS, pH 7.4. Forty µl of 1.0 mg/ml lipoproteins were injected. Fractions were assayed for Lp-PLA_2_ activity as described in Experimental Procedures. (**g**) Fractionation of human serum with and without treatment with 150 µM darapladib under the conditions as indicated by TSKgel G3000SW_XL_ column in TBS, pH 7.4. Forty µl of neat serum were injected. Fractions were assayed for residual Lp-PLA_2_ activity as described in Experimental Procedures.

The inhibitory effects of darapladib over Lp-PLA_2_ were analyzed under different conditions for LDL and HDL separately. Inhibition of lipases can be complicated and depends on how the inhibitors are delivered^22^. Figure 5 shows the responses of Lp-PLA_2_ bound on LDL and HDL in the presence of darapladib under different experimental conditions. When LDL and HDL were assayed for Lp-PLA_2_ activity by adding the mixture of 14:0 NPS-PC (final concentration of 10 µM) and varied concentrations of darapladib to start the reaction, only partial inhibition of the enzyme was achieved at the highest concentration of darapladib and the IC_50_ was about 0.5 and 1 nM for LDL and HDL respectively (Fig. 5a,b). If preincubating the LDL and HDL with darapladib at ambient temperature for 30 min before adding 14:0 NPS-PC to start the reaction, the IC_50_ was about 10 nM for both LDL and HDL. When 40 mM of CHAPS (final concentration in the assay mixture was 20 mM) were included in the preincubation mixture of darapladib and LDL or HDL, the IC_50_ was about 1 nM for both LDL and HDL. Also, higher activity and more complete inhibition of Lp-PLA_2_ were observed in the presence of CHAPS. For HDL, the associated Lp-PLA_2_ activity is relatively less sensitive to the inhibition by darapladib in the absence of CHAPS. The IC_50_ of darapladib for both LDL- and HDL-associated Lp-PLA_2_ was dependent on the concentration of LDL and HDL (Fig. 5c–e). When lipoprotein concentration was reduced 100-fold, Lp-PLA_2_ activity in LDL became much more sensitive to darapladib and was completely suppressed at less than 0.1 nM of darapladib. In the presence of 20 mM CHAPS, the IC_50_ of darapladib remained the same for both concentration levels of LDL and HDL (Fig. 5e). On the other hand, Lp-PLA_2_ activity associated with HDL was less sensitive to the decrease of lipoprotein concentration especially under the conditions where the lipoproteins were preincubated with darapladib (Fig. 5c,d). Such difference disappeared when CHAPS was included in the incubation mixture and the Lp-PLA_2_ activity in both LDL and HDL decreased significantly (Fig. 5e). The IC_50_ of darapladib for Lp-PLA_2_ was the same for both LDL and HDL and not affected by lipoprotein concentration in the presence of CHAPS. The resistance of HDL-bound Lp-PLA_2_ to darapladib was further confirmed by excessive inhibition of the enzyme in human serum with 150 µM of darapladib. After incubation with high concentration of darapladib and removal of the bulk content by size exclude chromatography (SEC) using a TSKgel G3000SW_XL_ (7.8 mm × 30 cm, 5 µm) in TBS, pH 7.4, the fractions of human serum were assayed for residual Lp-PLA_2_ activity. The column was calibrated with purified HDL and LDL in TBS, pH 7.4. LDL was eluted in fraction 7 and HDL was eluted in fractions 8–10 when 40 µl of HDL or LDL at the concentration of 1 mg/ml were injected for resolution (Fig. 5f). When the same volume of human serum was resolved by the same column under the same conditions, majority of Lp-PLA_2_ activity was fractionated in fraction 7 (Fig. 5g, blue). The distribution pattern did not change when the serum was incubated with 150 µM darapladib for 1 h at ambient temperature and fractionated (Fig. 5g, red). When the same serum-darapladib mixture was continued to incubate for 24 h at ambient temperature before fractionation, Lp-PLA_2_ activity in fraction 7 (LDL) disappeared completely indicating that the inhibitor was tightly bound. However, the Lp-PLA_2_ activity in the HDL fractions of the same mixture was intact and seemingly increased slightly (Fig. 5g, green). The same results were obtained when the experiment was repeated multiple times.

### Effects of detergent on the integrity of lipoproteins

**Figure 6:**
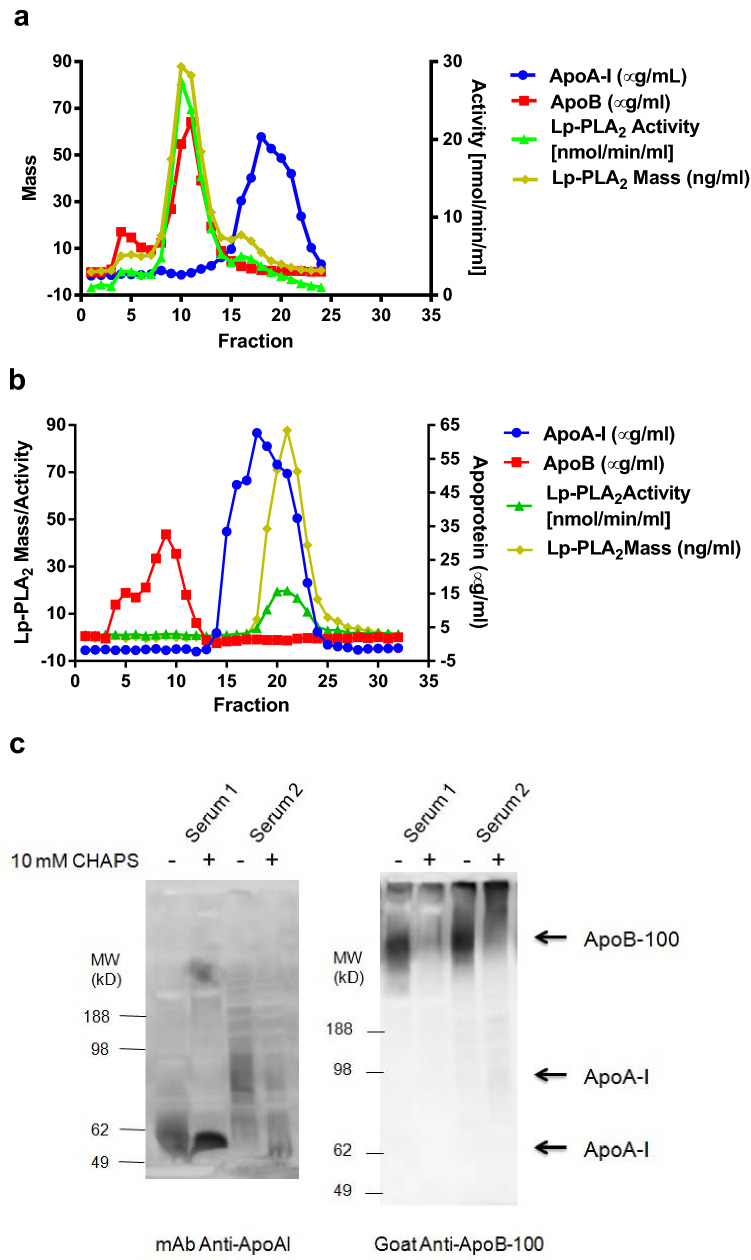
Effects of CHAPS on the integrity of lipoproteins. (**a**) Fractionation of human serum using Superose-6 column in the absence of detergents. (**b**) Fractionation of human serum using Superose-6 column in the presence of 10 mM CHAPS. (**c**) Non-denatured gel electrophoresis and Western blot of human sera (Serum-1 and Serum-2) treated with and without 10 mM CHAPS.

A serum from apparent healthy donor was fractionated by a Superose-6 column (10 × 300 mm) in the presence and absence of 10 mM CHAPS in phosphate buffered saline (PBS). The retention shifting of apolipoprotein A1 (ApoA1), apolipoprotein B (ApoB) and Lp-PLA_2_ were analyzed by ELISA and enzyme activity (Fig. 6). Consistent with earlier studies^11,12^, the majority of Lp-PLA_2_ mass (yellow curve) or activity (green curve) was associated with ApoB (LDL, red curve) and eluted in fraction 8–14 (Fig. 6a). A small portion of the enzyme was associated with ApoA1 (blue curve) and eluted in fraction 15–19 (larger size HDL, Fig. 6a). No bulk phase distribution of Lp-PLA_2_ activity or mass was found. When including 10 mM CHAPS in the fractionation buffer, however, all of the Lp-PLA_2_ mass and activity was dissociated from LDL and HDL (Fig. 6b). The peak fraction of ApoB was shifted from fraction 11 to fraction 9 in the presence of 10 mM CHAPS while no significant changes were observed for the elution pattern of ApoA1 under the same conditions. Similar results were obtained when serum samples were analyzed by non-denatured gel electrophoresis for ApoA-1 and ApoB-100 (Fig. 6c). Mixing serum with 10 mM CHAPS resulted in the aggregation of ApoB but less damage of HDL was observed except for some de-lipidation of ApoA1.

### Transfer of Lp-PLA_2_ between LDL and HDL

**Figure 7:**
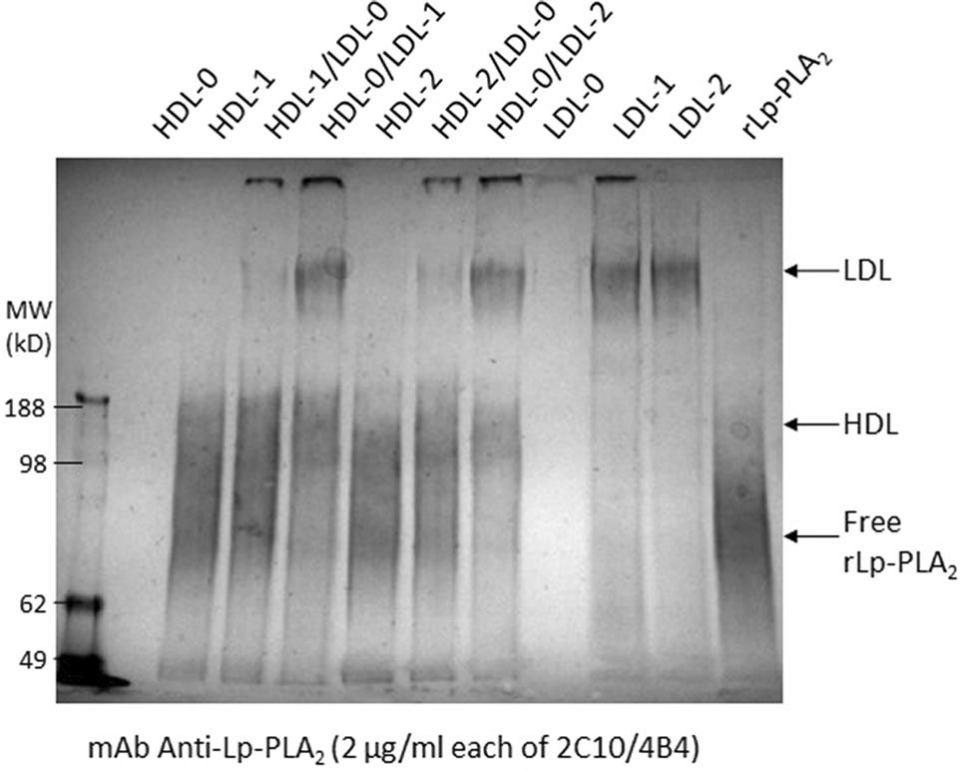
Transport of spiked recombinant Lp-PLA_2_ (rLp-PLA_2_) between purified HDL and LDL. Endogenous Lp-PLA_2_ were depleted from purified LDL and HDL by incubation with magnetic beads coupled with anti-Lp-PLA_2_ antibodies and separation of the supernatants. The Lp-PLA_2_ depleted LDL and HDL (LDL-0 and HDL-0) were spiked in duplication with 25 µg/ml of rLp-PLA_2_ and incubated at ambient temperature for 16 h (LDL-1, LDL-2, HDL-1 and HDL-2). LDL and HDL spiked with Lp-PLA_2_ were further mixed with blank HDL (HDL-0) or LDL (LDL-0) respectively as shown. All mixtures were further incubated at ambient temperature for additional 6 h and stored in 2–8 ºC for 16 h before subjected to resolution by non-denatured gel electrophoresis as described in Experimental Procedures.

Lp-PLA_2_-free LDL and HDL were prepared from normal human sera by incubation of the purified lipoproteins with magnetic beads labeled with anti-Lp-PLA_2_ monoclonal antibody and removal of the Lp-PLA_2_ carrying lipoproteins. Both the Lp-PLA_2_-free LDL and HDL (Lane LDL-0 and HDL-0 in Fig. 7) were shown to have the capacity of binding exogenous recombinant Lp-PLA_2_ spiked into the mixtures (Lane LDL-1, LDL-2, HDL-1 and HDL-2 in Fig. 7). Exchange of Lp-PLA_2_ between LDL and HDL was studied by mixing and incubation of the two lipoproteins, in which one or the other was spiked with recombinant Lp-PLA_2_. The mixed lipoproteins were analyzed by non-denatured gel electrophoresis and Western blotting using anti-Lp-PLA_2_ antibody. Figure 7 shows that Lp-PLA_2_ loaded on LDL can be transferred to HDL during mixing of the two lipoproteins (Lane HDL-0/LDL-1 and HDL-0/LDL-2 in Fig. 7). However, very low levels of Lp-PLA_2_ loaded on HDL can be transferred to LDL during the mixing (Lane HDL-1/LDL-0 and HDL-2/LDL-0 in Fig. 7).

### Distribution of darapladib and 14:0 NPS-PC in bulk phase, detergent micelle, lipoprotein and human serum

**Figure 8:**
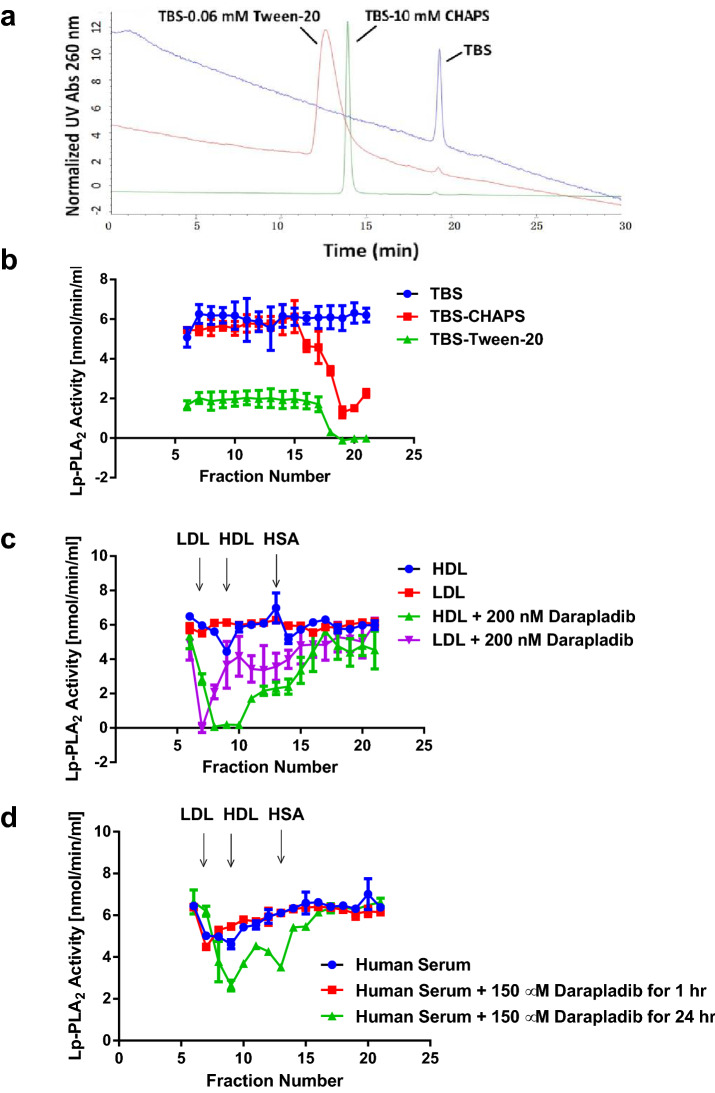
Distribution of 14:0 NPS-PC and darapladib under different conditions. Assay methods are described in Experimental Procedures. (**a**) Distribution of 14:0 NPS-PC in TBS, pH 7.4, with and without detergents. Twenty µl of 14:0 NPS-PC at 10 µM were resolved by a TSKgel G3000SW_XL_ column in TBS, pH 7.4, containing no detergent, 10 mM CHAPS or 60 µM Tween-20. Absorbance at 260 nm were monitored for 14:0 NPS-PC. Bulk phase is after fraction number 17 or after elution time 17 min. (**b**) Distribution of darapladib in TBS, pH 7.4, with and without detergents. Forty µl of darapladib in mobile phase buffer at 0.2 µM were resolved by a TSKgel G3000SW_XL_ column in TBS, pH 7.4, containing no detergent, 10 mM CHAPS or 600 µM Tween-20. Relative darapladib concentration in each fraction was shown by inhibition of spiked Lp-PLA_2_. (**c**) Distribution of darapladib in TBS, pH 7.4, containing HDL or LDL. Forty µl of 1 mg/ml HDL or LDL with or without 0.2 µM darapladib were resolved by a TSKgel G3000SW_XL_ column in TBS, pH 7.4. Relative darapladib concentration in each fraction was quantitated by inhibition of spiked Lp-PLA_2_. (**d**) Distribution of darapladib in human serum. Forty µl of neat human serum with or without 150 µM darapladib under the conditions as indicated were resolved by a TSKgel G3000SW_XL_ column in TBS, pH 7.4. Relative darapladib concentration in each fraction was determined by inhibition of spiked Lp-PLA_2_.

To interpret the results of activation and inhibition of Lp-PLA_2_, it is important to know the distribution of darapladib and 14:0 NPS-PC in bulk phase, detergent micelles and lipoproteins. Distribution of 10 µM 14:0 NPS-PC in bulk (TBS, pH 7.4) and micelles of two represent detergents, CHAPS (charged and higher CMC) and Tween-20 (non-charged and lower CMC), was investigated by separation of the solutions using the same TSKgel G3000SW_XL_ column. As indicated in Fig. 8a, 10 µM 14:0 NPS-PC existed only as monomer in the bulk phase at the absence of detergents and almost completely (> 95%) formed mixed micelles with the presence of 10 mM CHAPS or 60 µM Tween-20. Less than 5% of monomeric 14:0 NPS-PC was detected in bulk in the presence of detergents under the conditions. Distribution of 14:0 NPS-PC in lipoprotein particles was not determined due to technical difficulties. Figure 8b shows the distribution of darapladib in TBS, pH 7.4, in the absence and presence of detergents. Since the concentration of darapladib was low (0.2 µM) and not easy to monitor, fractions were collected and assayed for its ability to inhibit spiked Lp-PLA_2_. The results indicated that, in the absence of detergents, darapladib at 0.2 µM was not detectable in TBS buffer, possibly due to the low solubility (Fig. 8b, blue). The compound was solubilized in the presence of either 10 mM CHAPS (Fig. 8b, red curve) or 0.6 mM Tween-20 (Fig. 8b, green curve). However, it eluted in very late fractions possibly due to the interaction of darapladib-detergent complex with the column packing materials. When mixtures of darapladib with HDL or LDL were fractionated, inhibition of spiked Lp-PLA_2_ was observed with the fractions of the lipoprotein correspondingly (Fig. 8c). Darapladib only associated with LDL, HDL and albumin and was not present in bulk phase under the experimental conditions. Figure 8d shows the distribution of darapladib in human serum and further confirms the association of darapladib with HDL and albumin. Darapladib was mixed with human serum at the final concentration of 150 µM and incubated at ambient temperature as indicated. Forty µL of the mixture were subjected to fractionation by the same TSKgel G3000SW_XL_ column. Inhibition of spiked Lp-PLA_2_ was only observed with the HDL and albumin fractions (Fig. 8d, green curve). Again, no bulk distribution for darapladib was found. Also, darapladib inhibition of spiked Lp-PLA_2_ was not observed in the LDL fraction (fraction 7, green curve in Fig. 8d) albert the endogenous Lp-PLA_2_ activity of LDL was completely inhibited as shown in Fig. 5g.

## Discussion

Lipases are enzymes that catalyze the hydrolysis of lipids at the ester bond and belong to a subclass of esterases. However, the main difference between regular esterases and lipases is that esterases prefer substrates in solution and lipases prefer substrates at the phase interfaces^23^. Like other lipases, Lp-PLA_2_ catalyzes the hydrolysis of phospholipids at the lipid-water interfaces, involving interfacial adsorption and subsequent catalysis. Catalytical reactions in Fig. 1a demonstrate that Lp-PLA_2_ has low hydrolytic activity when the concentration of artificial substrate,14:0 NPS-PC, at 10 µM is too low to form aggregates or micelles. Figure 8a shows that 14:0 NPS-PC elutes from the TSKgel G3000SW_XL_ column as monomers at 10 µM. Titration of four detergents with different critical micelle concentration (CMC) results in the stimulation of Lp-PLA_2_ hydrolytic activity around the CMC of each detergent. This correlation strongly suggests that the formation of water-micelle interface is critical for the activation of Lp-PLA_2_. On the other hand, the CMC of 14:0 NPS-PC is found to be near 0.5–1 mM (Fig. 3c). A plot of the reaction rate against the substrate concentration in the presence and absence of 10 mM CHAPS shows that the kinetics of the reaction are different under the two situations. While the reaction is typical Michaelis–Menten kinetics with Hill slope of 1.0 in the presence of 10 mM CHAPS, it becomes a positively cooperative sigmoidal curve with Hill slope of 2.0 in the absence of detergent. In the absence of detergent, the reaction is significantly accelerated at substrate concentration above 20 µM (log1.3, Fig. 3a) which is much lower than its determined CMC, 0.5–1.0 mM (Fig. 3c). This suggests that Lp-PLA_2_ can hydrolyze monomeric or low aggregated form of 14:0 NPS-PC without the formation of micelles. Higher Hill slope in the absence of detergents indicates that 14:0 NPS-PC probably needs first to bind to the regulatory site of Lp-PLA_2_ and to change its conformation before the enzyme can bind and hydrolyze the substrate at the active site (similar to interfacial activation)^24^. This leads to the positive substrate cooperation when concentrations of 14:0 NPS-PC are lower than 0.5 mM and explains the deviation of Michaelis–Menten mechanism in the absence of detergents (substrate activation instead of interfacial activation)^25^. When concentration of 14:0 NPS-PC reaches its CMC, the Hill slope of the hydrolytic reaction decreases to 1.0 due to the formation of substrate micelles. Detergents play roles in the formation of aggregate and activation of Lp-PLA_2_ so that the enzyme can bind and hydrolyze the substrate. Since the enzyme has been activated in the presence of detergents, the Hill slope remains constant at 1.0 in all substrate concentration range (Fig. 3b). This supports the model first proposed by Wieloch et al*.* that there is a hydrophobic region in lipases which regulates the activation of the enzyme and is different from the enzyme active site^26,27^. Several decades of extensive studies have built models for interfacial activation of various PLA_2_^28,29^. Interfacial activation of certain enzymes involves conformational changes or displacement of a lid which covers the active or catalytical site slot, while the others become activated via yet unidentified mechanisms^28^. It is generally in common that the membrane or a hydrophobic surface acts as an allosteric ligand, shifting the conformation of a PLA_2_ from the closed form in water to the open form on the surface of the membrane or hydrophobic aggregate^29^. This process enables the enzyme to bind a phospholipid molecule in the active site (ES·M), where it is converted into product (EP·M)^29^. Figure 8a demonstrates that 14:0 NPS-PC forms mixed micelles with CHAPS or Tween-20, two represented detergents in the study, and has very low level of distribution in the bulk phase. Lp-PLA_2_ has been shown to have the similar distribution in detergents^12^. Lp-PLA_2_ may extract substrate from bulk phase, another micelle or the same micelle where it resides. If Lp-PLA_2_ extracts substrate within the same micelle, bringing enzyme and substrate into close approximate is also an important factor to accelerate the reaction by detergents.

Unlike classical interfacial activation, further increase of detergent concentration does not enhance, but rather inhibits, the Lp-PLA_2_ lipase activity. Lineweaver–Burk plot of the inhibition by Tween-20 reveals the competitive mechanism between substrate and detergent (Fig. 1c). Comparing the Michaelis–Menten kinetic parameters in the presence and absence of 10 mM CHAPS confirmed the competitive mechanism because competitive inhibition only affects K_m_ and not V_max_^25^. The hydrophobic regulatory site of Lp-PLA_2_ binds not only to the surface of detergent micelles but also to the surface of polystyrene latex beads. The interfacial activation can also be induced when Lp-PLA_2_ binds to the surface of polystyrene latex nanobeads with diameter of 100 nm or smaller (Fig. 1e). Higher concentrations of polystyrene latex nanobeads also turn to inhibition of the Lp-PLA_2_ activity (Fig. 1e). The smaller polystyrene nanobeads are slightly more potent in both the activation and inhibition of Lp-PLA_2_ probably due to higher particle number (at 0.1% w/w, 25 nm beads = 1.2 × 10^14^ particles/ml, 50 nm beads = 1.4 × 10^13^ particles/ml and 100 nm beads = 1.8 × 10^12^ particles/ml). Negatively charged polystyrene beads are more potent than the same size beads without charges. These phenomena support that the interfacial Lp-PLA_2_ probably extracts substrates from bulk phase or different micelles because nanobeads can absorb Lp-PLA_2_ on surface but contain no substrate. Therefore, surface dilution^30^ cannot explain the inhibition. That carboxylated latex beads have better binding affinity for Lp-PLA_2_ suggests that the hydrophobic binding site on the enzyme composes of hydrophobic and cationic residuals and therefore prefer the binding of negatively charged amphiphiles. This is similar with other PLA_2_^28^. The same pattern in activation and inhibition of recombinant Lp-PLA_2_ is also observed for HDL and LDL which are devoid of endogenous Lp-PLA_2_ (Fig. 1f). Based on the results of our studies, it can be proposed that binding of Lp-PLA_2_ onto the membrane or a hydrophobic surface stabilizes the substrate binding domain. The substrate binding structure of Lp-PLA_2_ must be quite open in order to accommodate lipid aggregate or cell membrane, from which phospholipids can be extracted or diffuse to the catalytical site of the enzyme. High concentration of detergents, polystyrene nanobeads or lipoprotein particles may fill in the substrate binding structure and block the access of substrates to the catalytical site resulting in inhibition of the enzyme. This explains why materials with hydrophobic surface such as detergent micelles, lipoprotein particles or polystyrene latex nanobeads can be both activator and inhibitor for Lp-PLA_2_ in a concentration dependent manner.

Earlier studies in our laboratory have shown that Lp-PLA_2_ has a hydrophobic binding site which can be disrupted and cause collapsing of the enzyme structure if exposed to the aqueous milieu^31^. Thus, monomeric Lp-PLA_2_ is unstable in aqueous bulk phase without substrates and therefore forms self-aggregates. Binding of Lp-PLA_2_ to lipoproteins or other hydrophobic surfaces protects the enzyme from denaturation in addition to the activation of the enzyme^31^. In human circulation system, Lp-PLA_2_ mainly associates with and is stabilized by LDL or HDL in an active conformation. This means that the substrate binding domain is in open conformation. What are the physiological substrates for Lp-PLA_2_ is not completely clear. However, from our data, the concentration of lipoprotein particles may affect the access of substrates to the active site of Lp-PLA_2_ because they can block the substrate binding domain of the enzyme and act as inhibitors. Lipoprotein particles or detergent micelles not only block the access of substrates but also block the access of inhibitors such as darapladib as demonstrated in Fig. 1d. In Fig. 5a,b, Lp-PLA_2_ is shown to be more sensitive to darapladib when the inhibitor is added with substrate together. If darapladib is preincubated with the lipoproteins before the addition of substrate, the inhibitory effect is reduced about ten-fold. The IC_50_ of darapladib is between that of the above situations when the lipoproteins are preincubated with darapladib in the presence of 40 mM CHAPS. Under the experimental conditions, concentration of Lp-PLA_2_ is in low double digit nM concentration range^32^ and the substrate 14:0 NPS-PC is 10 µM. Darapladib is at 0.1 to 200 nM. One mg/ml of LDL is about 1.1 µM and one mg/ml of HDL is about 67.3 µM of particles according to NMR studies^33^. Because the concentrations of Lp-PLA_2_ and darapladib are much lower than that of lipoprotein particles or detergent micelles, the odds for Lp-PLA_2_ to meet substrate or inhibitor can be affected by many different factors such as preincubation and lipoprotein particle numbers, as well as the affinities of substrate and inhibitor to lipoprotein particles as shown in Fig. 5. At 1 mg/ml concentration, HDL has much higher particle concentration (67.3 µM) than that of LDL (1.1 µM) and, therefore, darapladib is less inhibitory to HDL-associated Lp-PLA_2_ (Fig. 5b,d) because it is more diluted by HDL. As shown in Fig. 5d, inhibition of LDL Lp-PLA_2_ by darapladib was enhanced when the lipoprotein concentration was reduced 100-fold. However, reducing HDL concentration by 100-fold made no difference because HDL particle concentration was still much higher than that of darapladib. Furthermore, LDL-bound Lp-PLA_2_ may have higher affinity to darapladib than HDL-bound Lp-PLA_2_. While majority of darapladib is associated with HDL (Fig. 8d) and majority of Lp-PLA_2_ is associated with LDL (Fig. 6a), LDL-bound Lp-PLA_2_ activity can be completely inhibited but HDL-bound Lp-PLA_2_ activity remains intact when human serum is incubated with excess concentration of darapladib and subjected to separation by size exclusion chromatography. It is interesting to note that HDL-bound Lp-PLA_2_ is the indication of cardiovascular hazards but LDL-bound Lp-PLA_2_ may be not^12,32^ although the facts are difficult to be accepted by the research community due to that HDL is considered as “good” cholesterol. If HDL-bound Lp-PLA_2_ does play roles in cardiovascular diseases, resistance of HDL-bound Lp-PLA_2_ to darapladib may also contribute to the failure of the clinical studies.

Lp-PLA_2_ bound on HDL or LDL is already in activated state and can also be inhibited by detergents (Fig. 2). When lipoproteins are treated with detergents, Lp-PLA_2_ is usually extracted into detergent micelles (Fig. 6b and reference^12^). Also, darapladib is more soluble in the presence of detergents. These explain why darapladib has better inhibitory effect when lipoproteins are preincubated in the presence of CHAPS which forms micelles and improves the solubility of darapladib. Aggregation of ApoB indicates that LDL may be stripped off phospholipids by detergent micelles and collapsed (Fig. 6). However, majority of HDL seems still intact (Fig. 6) indicating that it is partially resistance to detergents. Preincubation of lipoproteins with darapladib in the absence of detergents is more closely resemble to in vivo physiological conditions. Under these conditions, darapladib may have limited inhibitory effects on HDL Lp-PLA_2_ as shown in Fig. 5b,d,g. The reason why darapladib is more effective when delivered with the substrate of Lp-PLA_2_ together is not clear. Mixing darapladib with 14:0 NPS-PC may improve the solubility of darapladib and therefore increases the inhibitory effects. The in vitro Lp-PLA_2_ activity assays used in clinical studies to assess the residual Lp-PLA_2_ activity usually includes detergents or does not preincubate serum samples with darapladib. Therefore, the in vitro Lp-PLA_2_ activity assays used in clinical studies may overestimate the in vivo suppression of Lp-PLA_2_ activity.

Lp-PLA_2_ has been shown mainly associating with LDL and HDL in an average ratio about 3:1 respectively^10–13^. In our experiments, Lp-PLA_2_ is only detected to transfer from LDL to HDL but not from HDL to LDL (Fig. 7). This suggests that Lp-PLA_2_ on HDL may come from LDL and the transfer process is probably regulated because majority of Lp-PLA_2_ is still distributed on LDL despite the fact that HDL particles out-number LDL particles. Darapladib has higher affinity with HDL and albumin, which carry less or no Lp-PLA_2_ (Fig. 8d). The difference in distribution of Lp-PLA_2_ and darapladib suggests that low efficacy of the drug can be expected. There may be a possibility that darapladib induces the migration of Lp-PLA_2_ from LDL to HDL as shown in Fig. 5g. However, further studies are necessary to confirm.

In summary, Lp-PLA_2_′s substrate binding site (active site) is modulated by a regulatory site which has affinity towards hydrophobic surfaces of membrane or aggregates. First, the hydrophobic surfaces of membrane or aggregates bind to the regulatory site of the enzyme and act as an allosteric ligand to change and stabilize the conformation of the active site (interfacial activation). Subsequently, it promotes the substrate binding on to the active site and facilitates the catalysis. Lp-PLA_2_ also hydrolyzes high concentration (> 20 µM but < 0.5 mM) of the artificial substrate, 14:0 NPS-PC, in solution without interfacial activation by hydrophobic surfaces of membrane or aggregates. However, kinetics of the reaction is positively cooperative with Hill slopes > 1.0 under such conditions, possibly due to that the monomeric or lamellar substrate acts as the allosteric ligand to bind on the regulatory site of the enzyme. The positively cooperative Hill slopes decrease as the concentrations of the phosphocholine substrate increase and reach to 1.0 when substrate micelles form around the CMC of 14:0 NPS-PC (0.5–1 mM). Detergent micelles, polystyrene latex nanoparticles or lipoprotein particles not only activate Lp-PLA_2_ by binding onto the regulatory site but also inhibit Lp-PLA_2_ by blocking the active site. Blocking the active site by lipoproteins in circulatory system actually protects Lp-PLA_2_ from inhibition by potent inhibitors such as darapladib. Darapladib has very low solubility and mainly associates with HDL and albumin. High concentration of lipoproteins will dilute the in vivo concentrations of darapladib and result in the efficacy variability for patients. Furthermore, In vitro activation and inhibition of Lp-PLA_2_ are very much dependent on the buffer components because it affects the affinities of enzyme to inhibitors and the availability of the inhibitors. In vitro Lp-PLA_2_ activity assays usually involve detergents and therefore may mislead the estimation of the in vivo efficacy of drugs due to that detergent micelles can extract the lipoprotein-associated enzymes from lipoprotein particles. Preincubation of lipoprotein particles with darapladib more closely resembles to the in vivo conditions but shows much less effective for darapladib in the inhibition of Lp-PLA_2_. Although darapladib mainly associated with HDL and albumin, HDL-associated Lp-PLA_2_ has lower affinity to bind the inhibitor. It is also worth to note that HDL-associated Lp-PLA_2_ predicts CVD events but LDL-associated Lp-PLA_2_ does not correlate with the diseases^12,32^. These factors together could cause darapladib to be less effective as expected especially for patients with high lipoprotein levels who actually are the population of high risk for cardiovascular diseases and it could be one of the reasons for the failure of clinical studies. If Lp-PLA_2_ does play roles in the development of cardiovascular diseases, the efficacy of darapladib might be better seen in the population of lowest lipoprotein level at the highest drug dosage. All of these assumptions do not consider the effects of lipoprotein remodeling, metabolism and other factors in vivo.

## Experimental procedure

### Materials

1-myristoyl-2-(4-nitrophenylsuccinyl)-sn-glycero-3-phosphocholine (14:0 NPS-PC) was purchased from Avanti Polar Lipids, Inc. (Alabaster, AL). The 10 × 300 mm Superose-6 column was manufactured by GE Healthcare Life Sciences (Piscataway, NJ) and the TSKgel G3000SW_XL_ (7.8 mm × 30 cm, 5 µm) was manufactured by Tosoh Bioscience (Griesheim, Germany). Purified LDL and HDL were obtained from Lee BioSolutions (St. Louis, MO). CHAPS, Triton X-100, digitonin, Tween-20, SNS, PBS and TBS were purchased from Sigma-Aldrich (St. Louis, MO). Criterion XT Tris–Acetate Precast Gels and reagents for non-denatured electrophoresis were purchased from Bio-Rad (Hercules, CA). Rabbit anti-Lp-PLA_2_ polyclonal antibodies were originally obtained from GlaxoSmithKline and also purchased from Cayman Chemicals (Ann Arbor, MI). Anti-rabbit second antibody-HRP conjugate was from Jackson ImmunoResearch Laboratories (West Grove, PA). Western plot detection kits and Dynabeads Antibody Coupling Kit were from Thermofisher. 2C10 and 4B4 antibody-coupled Dynabeads were prepared by following manufacturer’s protocol. Latex polystyrene beads were from Seradyn (Indianapolis, IN), Phosphorex (Hopkinton, MA) and Applied Physics, Inc. (Monte Vista, CO). PLAC mass and activity test kits for the quantitation of Lp-PLA_2_ are the products of diaDexus Inc. Recombinant Lp-PLA_2_, monoclonal antibodies against human Lp-PLA_2_, 2C10 and 4B4, were also made by diaDexus Inc. as the components of PLAC test kit. Human sera and plasmas were obtained as archival or fresh samples from a commercial vendor, ProMedDx (Norton, MA) who collected blood samples from consented donors, who provided informed consent, under protocols approved by an Institutional Review Board (IRB) of participating facilities using standard operating procedures. Names of IRB were not revealed by the vendor but the protocols were reviewed and approved by Research Ethic Review Board of diaDexus Inc. Other equipment or reagents were indicated in the text.

### Non-denatured gel electrophoresis, Western blotting and protein concentration determination

Criterion XT Tris–Acetate Precast Gels (3–8% gradient, Bio-Rad, CA) were prerun for 30 min at 100 V in running buffer (25 mM Tris, 192 mM glycine & 2 mM EDTA, pH 7.4) according to the instruction of manufacturer. Upper reservoir was refilled with fresh buffer before loading. Ten µl of single vesicles and 20 µl of double vesicles or blank vesicles were mixed with sucrose to final concentration of 200 mg/ml and loaded. Gels were run at 100 V for 3 h and blotted on to nitrocellular membranes in a buffer of pH 7.5, containing 25 mM Bicine, 25 mM Bis–Tris, 1 mM EDTA and 0.05 mM chlorobutanol at 50 V for 1 h. Western blots were analyzed by using rabbit anti-Lp-PLA_2_ polyclonal antibody or as indicated in the figures. Pierce Fast Western Blot Kit was used for detection and manufacturer’s protocol was followed. All protein concentrations were determined by using either micro BCA or modified Bradford protein assays (Pierce Biotechnology) following the manufacturer’s protocols. Both assays gave similar results for recombinant Lp-PLA_2_.

### Lp-PLA_2_ activity and mass assays

Lp-PLA_2_ activity was assayed under different conditions and different methods were used. Interfacial activation and inhibition of Lp-PLA_2_ were assayed in TBS or PBS, pH 7.4–7.6, with 10 µM of 14:0 NPS-PC substrate and detergents, polystyrene latex nanobeads or Lp-PLA_2_-depleted lipoprotein particles. Purified recombinant Lp-PLA_2_ in storage buffer was diluted into TBS or PBS, pH 7.4–7.6, at the concentration of 1 µg/ml and 20 µl of the solution were used for each reaction. The reactions were started by the addition of 110 µl reaction buffers (TBS or PBS, pH 7.4–7.6, containing 11.9 µM 14:0 NPS-PC and detergents, latex nanobeads or Lp-PLA_2_-depleted lipoproteins as indicated in figures) into each well of a 96-well plate containing the diluted enzyme. Generally, hydrolysis of 14:0 NPS-PC was monitored at 405 nm for 5 min under ambient temperature for all kinetic assays. Inhibition of Lp-PLA_2_ was carried out by adding 110 µl of 100 mM HEPES buffer, pH 7.4, containing 11.9 or 638 µM (final concentration 10 or 540 µM) of 14:0 NPS-PC substrate, 4 mM EDTA and different concentration of detergents or darapladib as indicated in figures to 20 µl of Lp-PLA_2_. For assays of endogenous Lp-PLA_2_ in lipoproteins, 20 µl of LDL or HDL (concentration as indicated in figures) were pre-incubated with 55 µl of assay buffer containing darapladib with or without 40 mM CHAPS (final concentration in assay 17 mM) for 30 min at ambient temperature. Assay were started by addition of 55 µl of 24 µM 14:0 NPS-PC in the same assay buffer (final concentration in assay 10 µM). Lp-PLA_2_ kinetic assays were performed in the same assay buffer with 14:0 NPS-PC concentrations as indicated. Purified Lp-PLA_2_ was about 5–25 ng/assay in volume of 5–20 µl or as indicated in figures. Lipoproteins and other assay conditions were as indicated in figures. Assays were carried out in 96-well plates using a SpectraMax m2e plate reader. Hydrolysis of 14:0 NPS-PC was monitored at 405 nm for 5 min under ambient temperature. Serum Lp-PLA_2_ activity and mass concentration were measured by using PLAC activity and mass test kits (diaDexus) following the manufacturer's instruction. PLAC mass measurements were performed by manual ELISA and PLAC activity measurements were carried out by the Beckman Coulter AU400 analyzer as described in a previous publication^12^. Twenty or forty μl of the fractionated sample per tube were used, depending on the Lp-PLA_2_ concentration of the samples. Ten mM CHAPS-modified PLAC ELISA test kits were made by adding solid CHAPS to the assay buffer to the final concentration of 10 mM. The assays follow the same instructions as the unmodified PLAC mass test kits. Other Lp-PLA_2_ activity assays are as indicated in figures or other experimental sections.

### Other assays

ApoA1 and ApoB mass concentrations were measured by using ELISA assay kits from AlerCHEK, Inc. (Portland, Maine), following the manufacturer's inserted instruction.

### Preparation of Lp-PLA_2_-depleted LDL and HDL lipoproteins

Concentrated human LDL and HDL were purchased from Lee BioSolutions in St. Louis. According to the manufacturer, LDL and HDL were prepared from fresh human plasma by undisclosed precipitation methods. Both the LDL and HDL showed one major band by Helena lipoprotein cellulose acetate electrophoresis. Characterization indicated that triglyceride/cholesterol ratios were 0.86 and 0.40 for LDL and HDL respectively. Concentration of LDL and HDL is indicated by that of cholesterol or triglyceride. Lp-PLA_2_-depleted LDL and HDL were prepared by incubation of lipoproteins with 2C10 and 4B4 antibody-coupled Dynabeads overnight at 2–8 °C and removal of supernatants. Alternatively, lipoproteins devoid of Lp-PLA_2_ were prepared as described before^31^. Briefly, the purchased lipoproteins were thawed and subjected to inactivation of Lp-PLA_2_ by incubation with 20 mM Pefabloc SC (Roche Applied Science, Indianapolis) in PBS, pH 7.2, at 2–8 ºC overnight. The Pefabloc SC inactivated lipoproteins were then dialyzed extensively with a 10 kD cutoff membrane in 1000-fold volume excess of buffer containing 50 mM phosphate, pH 7.2, and 150 mM sodium chloride with 3 exchanges at 2–8 ºC. The inactivated lipoproteins were found to have less than 10% of the original endogenous Lp-PLA_2_ activity by the PLAC activity assay. Both lipoproteins were further diluted to the desired concentrations before used in each experiment.

### FPLC and HPLC fractionation

Fractionation chromatography was carried out on an Akta10 or Akta100 by using a 10 mm × 300 mm Sperose-6 column at ambient temperature with the flow rate of 0.3 ml per minute. Fifty to two hundred µl of serum samples were injected per run depending on the Lp-PLA_2_ concentrations of the samples after the column was equilibrated with the running buffer (PBS, pH 7.2). Fraction collection was started at 21 min after sample injection and the collection volume was 0.6 ml/tube. Distribution of 14:0 NPS-PC and darapladib were carried out by separation of 20 – 40 µl of samples on a TSKgel G3000SW_XL_ (7.8 mm × 30 cm, 5 µm) in TBS, pH 7.4, with and without detergents using a HPLC at flow rate of 0.8 ml/min. Fractions were collected at 1 min per tube starting at 6 min and ending at 21 min. Signal of 14:0 NPS-PC was detected by monitoring at 260 nm. Darapladib were assayed based on its inhibition of Lp-PLA_2_ activity. To one hundred µl of fraction, 10 µl of Lp-PLA_2_ at the concentration of approximately 0.5 µg/ml were added and the mixture was incubated at ambient temperature for 30 min with shaking. Reaction was started by adding 50 µl of mixture of CHAPS, SNS (sodium 1-nonane sulfonate) and 14:0 NPS-PC in buffer of 150 mM HEPES, pH 7.5, containing 12 mM EDTA. The final concentrations of CHAPS, SNS and 14:0 NPS-PC were 5 mM, 10 mM and 50 µM respectively. The hydrolysis reaction was monitored at 405 nm for 5 min. Fractions were also assayed for residual Lp-PLA_2_ activity by mixing in 100 µl fraction with 50 µl buffer of 150 mM HEPES, pH 7.5, containing 30 mM SNS, 30 mM CHAPS, 12 mM EDTA and 1.6 mM 14:0 NPS-PC.

### Polarized fluorescence anisotropy

CMC of CHAPS and 14:0 NPS-PC were determined by polarized fluorescence anisotropy based on the method by Thorsteinsson et al*.*^21^ with modification in a Varian Cary Eclipse fluorescence spectrophotometer using fluorescein as probe. Fluorescein was dissolved in TBS, pH 7.4, at final concentration of 0.1 µM. Solid CHAPS (control) or 14:0 NPS-PC was dissolved in TBS-fluorescein solution at concentrations indicated in figure and subjected to serial dilution in the same buffer. All fleshly prepared solutions were measured for fluorescence emission at 520 nm (excited at 490 nm) and compared the emission optics for the horizontal orientation to the vertical orientation. The anisotropy ratio of fluorescence intensities was plotted against the concentration incrementation of CHAPS or 14:0 NPS-PC. The anisotropy ratio was calculated as the followings^34^.

$$ {\text{Anisotropy }}\,{\text{ratio}}\,{\text{ r }} = \, \left( {{\text{I}}_{{{\text{VV}}}} - {\text{ G }} \times {\text{ I}}_{{{\text{VH}}}} } \right) \, \div \, \left( {{\text{I}}_{{{\text{VV}}}} + \, 2 \, \times {\text{ G }} \times {\text{ I}}_{{{\text{VH}}}} } \right) $$

where I_VV_ indicates the intensity with vertically polarized excitation and vertical polarization on the detected emission. I_VH_ indicates the intensity when using a vertical polarizer on the excitation and horizontal polarizer on the emission. G is a grating factor used as a correction for the instrument’s differential transmission of the two orthogonal vector orientations.

### Reagent preparation

Darapladib was dissolved in dimethyl formamide (DMF) as 15 mM solution and stored at − 70 °C. The solution was further diluted in 50% isopropanol to 100 µM or 2 mM before mixing with buffers, HDL or LDL at the final concentration of 0.2 or 20 µM. For inhibiting human serum, darapladib in DMF was diluted in 50% isopropanol to 3 mM. To 5 µl of the 3 mM solution, human serum was added and mixed. The final concentration of darapladib was150 µM. 14:0 NPS-PC was dissolved in isopropanol or DMF at the concentration of 145 or 290 mM and stored at − 70 °C. The stock solution was directly diluted into buffers at varied concentration.

Experiment protocols related to human or animal samples were carried out in accordance with relevant guidelines and regulations. Methods were reviewed and approved by Research Ethical Review Board of diaDexus Inc.

## Abbreviations

14:0 NPS-PC: 1-Myristoyl-2-(4-nitrophenylsuccinyl)-sn-glycero-3-phosphocholine
CAD: Coronary artery diseases
CHAPS: 3-[(3-Cholamidopropyl) dimethylammonio]-1-propanesulfonate
CMC: Critical micelle concentration
DMF: Dimethyl formamide
EDTA: Ethylenediamine tetraacetic acid
ELISA: Enzyme-Linked Immuno Sorbent Assay
FPLC: Fast protein liquid chromatography
HDL: High density lipoprotein
HEPES: 4-(2-Hydroxyethyl)-1-piperazineethanesulfonic acid
HPLC: High Performance Liquid Chromatography
IC_50_: Half maximal inhibitory concentration
LDL: Low density lipoprotein
PBS: Phosphate buffered saline
SNS: Sodium 1-nonane sulfonate
TBS: Tris buffered saline
Tris: Tris(hydroxymethyl)aminomethane
